# Overcoming Gender Inequity in Prevention of Blindness and Visual Impairment in Africa

**DOI:** 10.4103/0974-9233.80695

**Published:** 2011

**Authors:** Herrieth Mganga, Susan Lewallen, Paul Courtright

**Affiliations:** Kilimanjaro Centre for Community Ophthalmology, Good Samaritan Foundation, Moshi, Tanzania

**Keywords:** Africa, blindness, cataract surgical coverage, gender

## Abstract

**Background:**

Globally, and in Africa, after adjusting for age, women are about 1.4 times more likely to be blind than men. While women generally live longer than men, the lack of accessibility to and use of services is likely the most important reason for excess blindness in women in Africa.

**Aim:**

We sought to review the literature on vision loss in Africa and summarize the findings related to gender equity.

**Materials and Methods:**

Information from across sub-Saharan Africa was collected on the evidence of gender inequity and reasons for this inequity. Finally, the results were used to generate suggestions on how gender equity could be improved.

**Results:**

In all published surveys (except one), cataract surgical coverage among women was lower than cataract surgical coverage among men. Although data available are limited, similar findings appeared in the use of services for other disease conditions, notably, childhood cataract and glaucoma. Evidence suggests that a variety of approaches are needed to improve the use of eye care services. Three main strategies are needed to address gender inequity in vision loss in Africa. First, it is important to address transport needs. Second, counseling of patients and family members is required. Finally, programs need to put in place pricing systems that make the services affordable the population.

**Conclusions:**

VISION 2020 can be achieved in Africa, but investment is needed in a variety of strategies that will ensure that eye care services are affordable, accessible, and acceptable to women and girls.

## INTRODUCTION

The WHO estimates that worldwide 37 million people are blind and an additional 124 million have some visual impairment.^1^ Most of these occur in the poorest economic regions. In developing countries, most people suffering with blindness lack awareness of the potential to cure the conditions, lack access to services, or do not accept services for a variety of cultural and social reasons. These problems prevent both men and women from availing the services they need but affect them differently.

A meta-analysis of population-based surveys on blindness prevalence in Asia, Africa, and the industrialized countries in 2000 indicated that women bear approximately two-thirds of the burden of blindness in the world.^2^ The excess blindness in women occurred in all the regions studied, but the factors behind the disparity vary by geopolitical regions. In industrialized countries, where age-related macular degeneration in most elderly is the main cause of blindness, the overall excess blindness in women is due to the fact that there are more nuber of elderly women than elderly men. In non-industrialized countries, the longevity in women contributes, but access to services is a major factor as well.

## EVIDENCE FROM AFRICA

The meta-analysis in 2000 showed that in Africa, specifically, the age-adjusted odds for women to be blind compared to men was 1.39 (95% CI: 1.20-1.61).^2^ Since then more population-based surveys have been conducted in Africa. Data from Rapid Assessment of Avoidable Blindness (RAAB) surveys usually show no statistically significant difference in blindness rates between men and women; however, these are not large enough to show expected differences according to sex. Two large population-based surveys in Africa showed a statistically higher prevalence of blindness (adjusted for age) among women compared to men. In Nigeria,^3^ women were 1.3 times (95% CI: 1.1-1.6) more likely to be blind compared to men, while in Ethiopia,^4^ the age-adjusted prevalence of blindness among women was 1.9% compared to 1.2% among men.

### Cataract

Cataract is the major cause of blindness in Africa and Cataract Surgical Coverage (CSC) is one of the most meaningful measures of service delivery for cataract. A meta-analysis of recent (since the year 2000) surveys, including CSC, found that males were 1.7 times (95% CI: 1.48-1.97) more likely to have received cataract surgery than women.^5^ The meta-analysis included seven studies from five countries in Africa (Kenya, Malawi, Nigeria, Rwanda, and Tanzania). In all but one study, CSC was higher in men compared to women [Table 1]. In Africa, if women received surgery at the same frequency as men, the prevalence of cataract blindness could be reduced by about 12%.

**Table 1:** Cataract surgical coverage (by individual, vision <6/60) in Africa

Country	Cataract surgical coverage (95% CI)	
	Women	Men
Kenya^29^	76.1 (67.9-84.3)	82.7 (75.2-90.2)
Kenya^30^	72.2 (60.2-81.8)	69.4 (57.3-79.5)
Malawi^31^	28.1 (12.5-43.7)	44.4 (25.7-63.1)
Nigeria^32^**	19.2 (6.1-32.2)	24.4 (9.8-39.0)
Rwanda^33^	34.5 (17.2-51.8)	55.6 (32.6-75.8)
Tanzania^34^	62.0 (52.5-71.5)	68.0 (58.6-77.4)
Tanzania^35^**	49.2 (39.7-58.7)	76.9 (68.8-85.0)*

* *P*<0.01,

** By individual, vision <3/60

Lower cataract surgical coverage among women is one of the contributing factors to the higher prevalence of blindness in women in Africa. Lower coverage in women is due to various social and cultural factors. A qualitative study conducted in Tanzania^6^ found that the perceived need for surgery varies by gender. Women are less likely to express a need for sight due to fear of being seen as a burden, and some household heads seem to be more inclined to support surgery for elderly men than elderly women.

### Trachoma

Trachoma, an important cause of blindness in some areas in Africa, is also more common in women than in men. A recent meta-analysis showed that women are 1.82 times (95% CI: 1.61-2.07) more likely to have trichiasis compared to men.^7^ A small study in South Sudan demonstrated that, even among children, girls were 1.5 times more likely than boys to have trichiasis.^8^ Many of the well-known risk factors for transmission of trachoma are related to gender,^9^ including caring for children, a task of women and girls. Once trichiasis develops, access to surgery becomes critical; evidence of inequity in the use of services is inconclusive with some settings showing equal utilizations,^10^ while others show lower use by women.^11^

### Childhood cataract

Cataract is one of the main avoidable causes of blindness in children, and it is estimated that worldwide, about 200,000 children suffer from blindness caused by cataract, with 20,000-40,000 born each year with congenital cataract.^12^ Surveys conducted in Malawi^13^ and Uganda^14^ showed that cataract is the single most important cause of blindness in children in these settings. Traumatic cataract is more common in boys than girls, but the incidence of congenital and developmental cataract was not significantly different in the only large study (in Denmark) to look at this question.^15^ Yet, in Tanzania,^16^ Kenya,^17^ and Senegal,^18^ almost two in three children receiving surgery for congenital or developmental cataract were boys. In the Tanzania study, girls with congenital cataract were brought later (mean age, 30.5 months) compared to boys (mean age, 20.2 months).^16^ Follow-up after surgery was also less frequent among girls compared to boys.^19^

In some African communities, when family resources are scarce, fathers and some mothers tend to give preference to boys as compared to girls.^20^ In addition, the decision-making capacity of mothers concerning the health and well-being of the family is often low.

### Glaucoma

Glaucoma is an important cause of blindness in Africa, although in most African countries, it has not been prioritized in VISION 2020 plans. When considering glaucoma and gender, Primary Open Angle Glaucoma (POAG) must be addressed separately from Primary Angle Closure Glaucoma (PACG). The latter is known in Asia and Europe to have an incidence 2-3 times higher in females than in males. Limited data from Africa are available, but there is some indication that it may be more common in African women than men.^21^

POAG is usually regarded as the most important of the glaucomas in Africa, and there is no evidence that it occurs more frequently in one sex than the other. There is, however, evidence that men receive surgery for glaucoma at twice the rate as women.^22^ This suggests underutilization of services by women.

## OVERCOMING GENDER INEQUITY IN BLINDNESS IN AFRICA

A number of strategies seem to help in decreasing the gender imbalance in service utilization in Africa.

### Addressing transport

Transportation in Africa is both expensive and difficult to use. The quality of roads and the dispersed nature of the population mean that people must often travel long distances to access care. Both long distances and poor quality roads result in transport costs often being too high for the rural poor to afford. This often affects women more because they have limited financial decision-making authority, limited funds of their own, and minimal experience in traveling outside of the village. The scenario applies for all major causes of blindness: cataract, glaucoma, trachomatous trichiasis, and childhood cataract.

Outreach programs, while expensive, are often the only way to reach rural populations, who otherwise would not have access to eye care services. Outreach that includes transport to the hospital for surgically treatable conditions provides the rural elderly with the physical and social capacity to seek care. Such systems also avoid unnecessary trips from one referral level to the next, which can be costly and discouraging for elderly patients. There is good evidence^23^ that outreach is most useful in reaching women.

Successful approaches to improving uptake of trichiasis surgical services have often relied on the surgical team to visit communities to provide trichiasis surgery. This also enables women to have easier access to these services. Generally, trichiasis surgeons who only work in static settings (like health centers or hospitals) have low levels of productivity.^24^

### Counseling

There are several social barriers that limit women from using eye care services, and approaches are needed to address each one of these. Social support, often lacking for women, can only be addressed by counseling of male members of the household—husbands and sons. One goal of counseling is to provide the family with the information necessary to make a decision that will benefit all concerned. A second goal of counseling is to ally fears that patients and family members may have regarding surgery.

Female peers in the community may be used to counsel and encourage families and women with visual problems to attend services. Through counseling, families are convinced to go for eye care services and fear of surgery is reduced. It must be recognized, however, that in many settings in Africa, even when cataract surgery is offered free of charge and transport is provided, not all patients (particularly women) are willing to accept surgery.^25^

Counseling is also important for parents of children who have congenital or developmental cataract. Research suggests that initial poor follow-up of girls who received surgery can be improved by provision of counseling (as well as cell phone contact) when the child is in the hospital.^26^ As parents have a significant role in ensuring that children return for follow-up, use their spectacles, and get appropriate placement, counseling often has to be done over a number of days. Thus, counseling is done at the time of admission, when the child is recovering from surgery, at a time of discharge, and during follow-up visits. Parents are encouraged to give equal opportunities to girls and boys.

### Setting a price for surgery within the family capacity to pay

Requiring patients to contribute to the cost of eye care services is important for improving financial sustainability in Africa. Charging for services tends to encourage greater accountability of service providers and leads to improved quality of care. On the other hand, if the amounts charged (including both direct and indirect fees) is too high, the price of service becomes a barrier.^27^ As women have limited financial decision-making authority in the family, cost of service often affects women disproportionately. Thus, determining the right price to charge (and considering indirect costs such as transport) that most families can afford, is a critical step in ensuring that eye care services are equitable. In Africa, due to a variety of factors (eg, low density of population, lack of a large middle class, large distances), achieving financial sustainability of eye care services is still a distant goal.^28^ This should not deter programs from setting reasonable fees and to educate the populace about the actual cost of providing eye care services.

## SUMMARY

Gender inequity in the use of eye care services remains a problem in Africa; however, understanding of the reasons for gender inequity have led some programs to explore ways to address the problem. While solutions will need to vary, country by country, setting by setting, there are some principles that apply across the continent. First, programs must address transport needs, particularly for the elderly (women) and children. For trichiasis surgery, outreach visits to communities to provide surgery may be required. Second, good counseling helps provide the information needed for better decision making and for education of the benefits of eye care (and follow-up). Third, setting prices for services within the capacity of the family to pay (and encouraging better financial decision making in the family) will improve service use for both women and men.

There is a need to understand “emerging” conditions in Africa—particularly glaucoma and diabetic retinopathy. The dearth of information on these two diseases has limited our understanding of how best to ensure equitable service delivery.

Even with the changes that have occurred in the past 10 years, problems and challenges remain. Most women still have limited decision making authority regarding healthcare and finances. Social support for elderly women to access services remains a problem in many settings. Finally, very few eye care programs in Africa monitoring service are disaggregated by sex, and fewer still have adopted gender-sensitive strategies. In the next 10 years, we anticipate that much can be done to address these challenges.
